# Identification of Specific Variations in a Non-Motile Strain of Cyanobacterium *Synechocystis* sp. PCC 6803 Originated from ATCC 27184 by Whole Genome Resequencing

**DOI:** 10.3390/ijms161024081

**Published:** 2015-10-12

**Authors:** Qinglong Ding, Gu Chen, Yuling Wang, Dong Wei

**Affiliations:** College of Light Industry and Food Sciences, South China University of Technology, 381 Wushan Road, 510641 Guangzhou, China; E-Mails: dingql21@126.com (Q.D.); wang.yulingwyl@163.com (Y.W.); fewd304@scut.edu.cn (D.W.)

**Keywords:** *Synechocystis* sp. PCC 6803, whole genome resequencing, single nucleotide polymorphism, deletion, insertion

## Abstract

Cyanobacterium *Synechocystis* sp. PCC 6803 is a widely used model organism in basic research and biofuel biotechnology application. Here, we report the genomic sequence of chromosome and seven plasmids of a glucose-tolerant, non-motile strain originated from ATCC 27184, GT-G, in use at Guangzhou. Through high-throughput genome re-sequencing and verification by Sanger sequencing, eight novel variants were identified in its chromosome and plasmids. The eight novel variants, especially the five non-silent mutations might have interesting effects on the phenotype of GT-G strains, for example the truncated Sll1895 and Slr0322 protein. These resequencing data provide background information for further research and application based on the GT-G strain and also provide evidence to study the evolution and divergence of *Synechocystis* 6803 globally.

## 1. Introduction

As the first sequenced photosynthetic organism and with high transformation competency, the freshwater cyanobacteria *Synechocystis* sp. PCC 6803 was one of the most widely used model organisms for the research in photosynthesis and stress response, as well as for the biotechnological application of biofuel production [1,2,3,4,5]. The original Berkeley strain of *Synechocystis* sp. PCC 6803 was isolated from freshwater in California [6] and deposited in the Pasteur Culture Collection as PCC 6803 strain and in the American Type Culture Collection as ATCC 27184 strain. A glucose-tolerant (GT) strain was isolated from ATCC 27184 and designated Williams GT strain [7], which later the GT-Kazusa strain was derived from. The chromosome sequences of GT-Kazusa were published as the first *Synechocystis* sp. PCC 6803 genomic sequence [8,9]. In recent years, based on the high-throughput sequencing techniques, several other strains of *Synechocystis* sp. PCC 6803 were sequenced and reported world widely [10,11,12,13]. Other than the database errors, unique sequence variations were identified in GT-S, GT-I, PCC-P (positive phototactic), PCC-N (negative phototactic) and PCC-M (Moscow, Russia) strain, as well as the GT-O1 and GT-O2 in New Zealand. It is suggested that strain-specific mutations are likely to be responsible for phenotypic variation, such as pilus biosynthesis and motility. Such widespread genomic variations imply that novel mutations may exist between and within research labs. Recent genomic analysis of stress-evolved *Synechocystis* sp. PCC 6803 strains also revealed interesting information in adaptive evolution and stress response under high temperature or low pH [14,15].

In our lab, a designated wild type strain of *Synechocystis* sp. PCC 6803 was originated from ATCC 27184 and subjected to mutant construction for analyzing the signal transduction in stress response [16,17,18,19]. It is glucose-tolerant [17], but its genomic background information was not defined. Thus we re-sequenced and analyzed our own wild type stain GT-G (Guangzhou, China) to provide reference information for future research and to clarify its phylogenetic relationships with various sequenced strains. Our results not only provide background information for further research and application based on GT-G strain, but also provide evidence to study the evolution and divergence of *Synechocystis* 6803 globally.

## 2. Results and Discussion

### 2.1. Overview

The glucose tolerant strain originated from ATCC 27184 through routine laboratory culture conditions in our lab was designated GT-G (Guangzhou) and subjected to genomic re-sequencing. More than 8 million short reads (101 bp per read) were obtained from Illumina Hiseq2000 sequencing platform, about 808 Mb high quality data in total. This represents more than 200 folds coverage of the 3.96 Mb *Synechocystis* 6803 chromosome and plasmid genome. Using BWA [20] and VarScan [21,22] software, genomic sequences were constructed and putative variants were identified through mapping reads to the reference sequence of GT-Kazusa chromosome and plasmids. SNPs and indels were identified, while no large structure variation was detected. The putative variants were then verified by Sanger sequencing of the corresponding PCR products. No false-positive variant was found. The genome sequence of GT-G was deposited in the GenBank database under the accession number CP012832.

In total, 40 SNPs and indels were identified and verified in GT-G strain, 34 in chromosome and six in plasmids (Table 1). Among these, 32 variations were previously reported, including the 21 database errors of GT-Kazusa reported previously [10] (Table 2). Excluding the errors of database, among the 19 mutations of GT-G, 10 mutations are shared with PCC-M, nine are shared with PCC-N and PCC-P, six are shared with GT-O1 and GT-O2, five are shared with GT-I, and three are shared with GT-S [10,11,12,13].

### 2.2. Chromosome Variations Shared with Other Strains

Mutation #1 implies that the 102 base pair deletion in *slr1084* is specific to the GT-Kazusa and GT-S strains (Table 2) [11,12]. Mutation #2 implies that GT-G originated from ATCC 27184 before the 154 base pairs deletion appeared upstream and within *slr2031*. However, GT-G shares with the other glucose tolerant, non-motile strains the 1 bp insertion in *sll1574/5* (*spkA*) gene, as checked and confirmed by PCR (Supplemental Table S1). The *spkA* gene was essential for motility and pilus biosynthesis [23,24] and its mutation might partly explain the non-motility in GT strain. Mutation #3 occurs in the non-coding region between *infA* and *adk* gene, 12 bp upstream of the transcriptional start site of *infA* gene [25]. This variation was also identified in PCC-P, PCC-N and PCC-M strain [11,12]. It changes the putative −10 element from “TGTGAT” to “TATGAT”. Thus it might have an effect on the transcription of *infA* gene, which encodes translation initiation factor IF-1.

It was reported that re-sequencing and mapping might fail to detect large indels, but report SNPs in the target region instead [11,13]. However in this study, several large indels are successfully called by mapping and confirmed by PCR and Sanger sequencing. Three 1.2 kb deletions in GT-G represent that the *ISY203b* (#6), *ISY203e* (#11), and *ISY203g* (#34) transposases insertion does not appear in GT-G, thus suggesting that they are specifically present in GT-Kazusa and/or GT-S [10,11,12].

The 1 bp deletion in *slr0162* (*pilC*, #14) is a variation common to all the reported PCC strains and GT strains except for GT-Kasuza [10], which suggests that the 1 bp insertion in *slr0162* was specific in GT-Kazusa (Table 2). This insertion caused a frameshift mutation in *pilC* gene and resulted in a truncated PilC protein, which might contribute to the lack of motility in GT-Kasuza [26]. Four novel variants in the chromosome unique to the GT-G strain are identified and verified as #8, #15, #16, and #17, which will be discussed in detail later. Two SNPs, mutations #27 and #29 are shared between GT-G strain and all PCC strains, suggesting their close relationship. They result in a silent mutation in PleD like protein coding gene *slr0302* and an amino acid change in a putative transposase ISY100v3 coding gene *ssr1176*, respectively.

### 2.3. Variations in Plasmids

Sequencing data cover all the seven plasmids and identify six mutations in three plasmids (#35–#40), which are all successfully verified by Sanger sequencing of PCR product. Of the six mutations in plasmid, four are unique to GT-G strain (#36, #37, #38, #40) and will be discussed in the next section. The 1.2 kb deletion in plasmid pSYSM (#35) represents the ISY203j transposase missing in GT-G, which was also reported in PCC-M [12]. The SNP in *ssr6089* of plasmid pSYSX (#39) results in a N37S change in the hypothetical protein, and is shared with GT-O1 and GT-O2 strains [13].

**Table 1:** Location and effects of SNPs and indels identified in GT-G compared with the nucleotide sequence of GT-Kazusa in the database. Specific variants identified in GT-G are highlighted in bold. The errors of database are in grey. SNP, insertions, deletions and intergenic region are labeled as S, I, D and IGR respectively.

Event							Effect		Locus		
#	M	Start	End	Size	Nucl Change	Ref→mut	AA Change	Result	Locus	Gene Name	Product
*Chromosome*											
1	I	386410	386411	102	-	-	-	34 additional AAs	slr1084	-	Hypothetical protein
2	I	781625	781626	154	-	-	-	5'extension of reading frame	IGR slr2030-slr2031	-	-
3	S	831647	831647	1	C→T	-	-	Possible effect on infA promoter	IGR adk-infA	-	-
4	S	943495	943495	1	G→A	GTC→ATC	V→I	AA 'change	slr1834	*psaA*	P700 apoprotein subunit Ia
5	S	1012958	1012958	1	G→T	-	-	-	IGR ssl3177-sll1633	-	-
6	D	1200294	1201476	1183	-	-	-	*ISY203b* missing	sll1780	-	Transposase
7	S	1364187	1364187	1	A→G	TTG→CTG	L→L	–silent-	sll0838	*pyrF*	Orotidine 5' monophosphate decarboxylase
**8**	**I**	**1765792**	**1765793**	**1**	*→T	**AAT→AAA**	**N→K**	**Frameshift**	**sll1895**	**-**	**Hypothetical protein**
9	S	1819782	1819782	1	A→G	TCT→TCC	S→S	–silent-	sll1867	*psbA3*	Photosystem II D1 protein
10	S	1819788	1819788	1	A→G	CTT→CTC	L→L	–silent-	sll1867	*psbA3*	Photosystem II D1 protein
11	D	2048412	2049594	1183	-	-	-	*ISY203e* missing	slr1635	-	Transposase
12	S	2092571	2092571	1	A→T	TTA→TAA	L→*	New stop codon	sll0422	-	Asparaginase
13	S	2198893	2198893	1	T→C	TTA→TTG	L→L	–silent-	sll0142	-	Probable cation efflux system protein
14	D	2204584	2204584	1	G→*	GGT→GTT	G→V	Frameshift	slr0162	*gspF,pilC*	A part of pilC, pilin biogenesis protein, required for twitching motility
**15**	**S**	**2235441**	**2235441**	**1**	**A→G**	**GGT→GGC**	**G→G**	**–silent-**	**sll1851**	**-**	**Hypothetical protein, no conserved domains**
**16**	**S**	**2272418**	**2272418**	**1**	**C→A**	**CCC→ACC**	**P→T**	**AA change**	**slr0322**	***pilL-C***	**Homologous to the C-terminal of CheA-like protein, essential for motility, thick pili biosynthesis and transformation competency.**
**17**	**D**	**2272927**	**2273907**	**981**	**-**	**-**	**-**	**delete 327 AAs**	**slr0322**	***pilL-C***	**As above**
18	S	2301721	2301721	1	A→G	AAG→GAG	K→E	AA change	slr0168	-	Hypothetical protein, no conserved domains
19	I	2350285	2350286	1	*→A	-	-	-	IGR sml0001-slr0363	-	-
20	I	2360245	2360246	1	*→C	GCG→GCC	A→A	Frameshift	slr0364/slr0366	-	Hypothetical protein, no conserved domains
21	D	2409244	2409244	1	C→*	GGA→GAT	G→D	Frameshift	sll0762	-	Hypothetical protein, no conserved domains
22	D	2419399	2419399	1	T→*	AAT→ATG	N→M	Frameshift	sll0751(ycf22);sll0752	*ycf22*	Hypothetical protein YCF22
23	I	2544044	2544045	1	*→C	AGG→GAG	R→E	Frameshift	ssl0787/ssl0788	-	Hypothetical protein, no conserved domains
24	S	2602717	2602717	1	C→A	CAC→CAA	H→Q	AA change	slr0468	-	Hypothetical protein, no conserved domains
25	S	2602734	2602734	1	T→A	ATT→AAT	I→N	AA change	slr0468	-	Hypothetical protein, no conserved domains
26	S	2748897	2748897	1	C→T	-	-	-	IGR slr0210-ssr0332	-	-
27	S	3014665	3014665	1	T→C	ACT→ACC	T→T	–silent-	slr0302	*pleD*	PleD-like protein
28	S	3096187	3096187	1	T→C	ATA→ACA	I→T	AA change	ssr1175(transposase)	-	Located in a mobile element(ISY100v1)
29	S	3098707	3098707	1	T→C	TGT→CGT	C→R	AA change	ssr1176(transposase)	-	Located in a mobile element(ISY100v3)
30	S	3110189	3110189	1	G→A	-	-	-	IGR sll0665-sll0666	-	Located in a mobile element(ISY523)
31	S	3110343	3110343	1	G→T	CCA→CAA	P→Q	AA change	sll0665	-	Transposase
32	S	3142651	3142651	1	A→G	CTT→CTC	L→L	–silent-	sll0045	*spsA*	Sucrose phosphate synthase
33	D	3260096	3260096	1	C→*	-	-	-	IGR sll0528-sll0529	-	-
34	D	3400331	3401513	1183	-	-	-	*ISY203g* missing	sll1474	-	Transposase
*pSYSM*											
35	D	117269	118451	1183	-	-	-	*ISY203j* missing	sll5131	-	Transposase
*pSYSX*											
**36**	**S**	**4241**	**4241**	**1**	**C→G**	**ATC→ATG**	**I→M**	**AA change**	**slr6004**	**-**	**Hypothetical protein, no conserved domains**
**37**	**S**	**4253**	**4253**	**1**	**C→T**	**CCC→CCT**	**P→P**	**–silent-**	**slr6004**	**-**	**Hypothetical protein, no conserved domains**
**38**	**S**	**4295**	**4295**	**1**	**T→C**	**TCT→TCC**	**S→S**	**–silent-**	**slr6004**	**-**	**Hypothetical protein, no conserved domains**
39	S	82405	82405	1	A→G	AAC→AGC	N→S	AA change	ssr6089		Hypothetical protein, no conserved domains
*pCB2.4*											
**40**	**D**	**1211**	**1211**	**1**	A→*	**CAG→CGG**	**Q→R**	**Frameshift**	**MYO_820**	**-**	**Hypothetical protein, no conserved domains**

**Table 2:** Comparison of SNPs and indels identified in GT-G with sequences from other reported strains. The errors of database are in grey. NI^a^: not investigated.

Variation		Strains Reported in Literatures and This Work								
#	Event	GT-Kazusa [10,11]	GT-S [10]	GT-I [11]	GT-O1 [13]	GT-O2 [13]	GT-G	PCC-P [11]	PCC-N [11]	PCC-M [12]
1	I	-	-	√	√	√	√	√	√	√
2	I	-	-	-	-	-	√	√	√	√
3	S	-	-	-	-	-	√	√	√	√
4	S	√	√	√	√	√	√	√	√	√
5	S	√	√	√	√	√	√	√	√	√
6	D	-	√	√	√	√	√	√	√	√
7	S	√	√	√	√	√	√	√	√	√
8	I	-	-	-	-	-	√	-	-	-
9	S	√	√	-	√	√	√	-	-	-
10	S	√	√	-	√	√	√	-	-	-
11	D	-	-	√	√	√	√	√	√	√
12	S	√	√	√	√	√	√	√	√	√
13	S	√	√	√	√	√	√	√	√	√
14	D	-	√	√	√	√	√	√	√	√
15	S	-	-	-	-	-	√	-	-	-
16	S	-	-	-	-	-	√	-	-	-
17	D	-	-	-	-	-	√	-	-	-
18	S	√	√	√	√	√	√	√	√	√
19	I	√	√	√	√	√	√	√	√	√
20	I	√	√	√	√	√	√	√	√	√
21	D	√	√	√	√	√	√	√	√	√
22	D	√	√	√	√	√	√	√	√	√
23	I	√	√	√	√	√	√	√	√	√
24	S	√	√	√	√	√	√	√	√	√
25	S	√	√	√	√	√	√	√	√	√
26	S	√	√	√	√	√	√	√	√	√
27	S	-	-	-	-	-	√	√	√	√
28	S	√	√	√	√	√	√	√	√	-
29	S	-	-	-	-	-	√	√	√	√
30	S	√	√	-	√	√	√	-	-	√
31	S	√	√	-	√	√	√	-	-	-
32	S	√	√	√	√	√	√	√	√	√
33	D	√	√	√	√	√	√	√	√	√
34	D	-	√	√	√	√	√	√	√	√
35	D	NI^a^	NI	NI	-	-	√	NI	NI	√
36	S	NI	NI	NI	-	-	√	NI	NI	-
37	S	NI	NI	NI	-	-	√	NI	NI	-
38	S	NI	NI	NI	-	-	√	NI	NI	-
39	S	NI	NI	NI	√	√	√	NI	NI	-
40	D	NI	NI	NI	-	-	√	NI	NI	-

### 2.4. Novel Variations in GT-G

Among the eight GT-G specific mutations identified here, five are SNP, two are deletion, and one is insertion, all of which locate in the open reading frame. Three SNPs (#15, #37 and #38) are silent mutations, while the other mutations cause amino acid change or frameshift.

**Figure 1:**
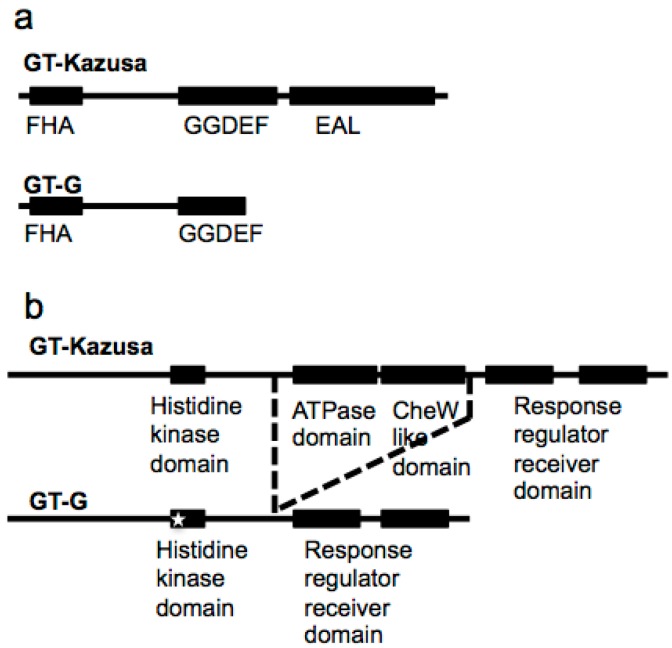
Domain organization of mutated genes coding protein in GT-Kazusa and GT-G. The black box indicates the conserved motif. (**a**) Domain organization of Sll1895. FHA: Forkhead-associated domain for phosphopeptide recognition; GGDEF: diguanylate cyclase domain; EAL: candidate for a diguanylate phosphodiesterase function. Sll1895 in GT-G loses EAL domain and part of the GGDEF domain; (**b**) Domain organization of Slr0322. The ATPase domain and CheW like domain are lost in GT-G, which is indicated by the dash lines. The white star indicates the P280T residue change in the histidine kinase domain in GT-G.

The 1 bp insertion in *sll1895* gene (#8) leads to frameshift and results in a truncated Sll1895 protein (Figure 1a). The 696 amino-acids long Sll1895 protein in GT-Kazusa is predicted to contain several functional domains, such as FHA (Forkhead-associated domain for phosphopeptide recognition), GGDEF (diguanylate cyclase domain), and EAL (candidate for a diguanylate phosphodiesterase function). It was suggested to contribute to signal transduction according to its conserved domain [27] and Sll1895 protein was found upregulated by hexane in a proteomic analysis [28]. The 377 amino acids-long truncated Sll1895 in GT-G strain lose EAL domain and part of the GGDEF domain, which may result in a non-functional protein (Figure 1a).

**Figure 2:**
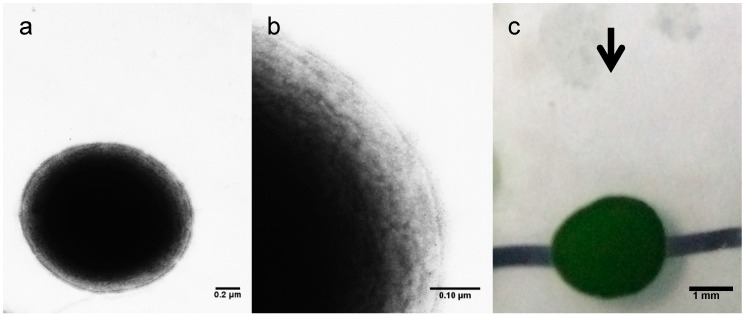
Surface structure and motility of GT-G. (**a**,**b**) Electron micrograph of negatively stained GT-G cells; (**c**) Phototactic movement of colonies of GT-G. The arrow indicated the direction of lateral light and the black line under the colony shows the initial position before lateral illumination.

A novel large deletion was revealed in GT-G as 981 bp deletion in the middle of *slr0322* gene (#17), resulting in 327 amino acids deletion inside the 1095 amino acids long Slr0322 protein (Figure 1b). Slr0322 in GT-Kasuza is a putative two-component hybrid sensor and regulator designated as Hik43, consisting of a histidine kinase domain and two response regulator domains in the N and C terminal respectively [3]. It was also designated PilL-C/CheA since it was homologous to the C-terminal of CheA-like protein and was essential for motility, thick pili biosynthesis, and transformation competency [29]. Slr0322 in GT-Kasuza strain contains the ATPase domain and CheW like domain between the kinase domain and response regulator domain, but they are lost in GT-G strain due to the deletion (Figure 1b). In addition, SNP in *slr0322* (#16) leads to a P280T residue change in the histidine kinase domain of this protein. Such functionally adverse mutations might have an effect on GT-G phenotype. Thus, we examined its surface structure under transmission electron microscope and its motility under lateral illumination. Electron micrographs of negatively stained GT-G cells indicated the deficiency of pilus and no phototactic movement of the GT-G colony was observed under lateral illumination (Figure 2). These phenotypes may be attributed to both the 1 bp insertion in *spkA* gene and the mutations in *slr0322*. Further research is needed to characterize the impact of individual mutations in GT-G strain.

Other than the two silent SNPs (#37, #38), SNP #36 in plasmid pSYSX is predicted to result in I64M change in unknown protein Slr6004. One base pair deletion in pCB2.4 (#40) leads to frameshift in hypothetical protein MYO_820 gene.

### 2.5. Phylogenetic Relationships

**Figure 3:**
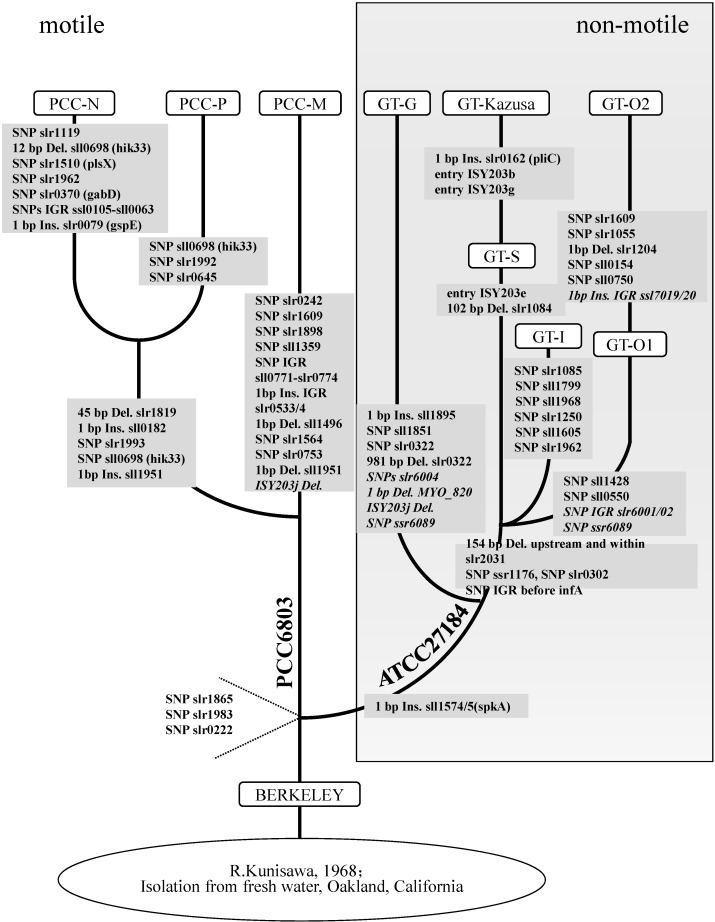
Visualization of phylogenetic relationships among various sequenced stains of *Synechocystis* sp. PCC 6803. The presence of identified SNPs and indels are indicated along the branches. The variants in plasmid are in italic. Putative insertions, deletions, and intergenic regions are labeled as Ins, Del, and IGR respectively. Modified after Trautmann *et al.*, Kanesaki *et al.* and Morris *et al.* [11,12,13].

Among the Synechocystis strains sequenced and reported so far, the genomic sequence of GT-G is most similar to PCC-M strain, sharing nine chromosome variants and one plasmid variant, though they are different in motile capacity (Table 2, Figure 2). According to our result and the published data, the phylogenetic relationships among various sequenced strains of *Synechocystis* sp. PCC 6803 are summarized in Supplemental Table S1 and visualized in Figure 3. GT-G strain can grow in glucose and cannot move towards light, which are characteristic of GT strains. GT-G strain shares with the other GT strains the 1 bp insertion in *sll1574/5* (*spkA*), which was critical for the motility and pilus biosynthesis [23,24]. However, it doesn’t contain the 154 bp deletion upstream and within *slr2031*, which makes it different from the other GT strains. GT-G shares with PCC strains SNP ssr1176, SNP slr0302 and SNP before infA, which suggests that GT-G may be the strain closest to the origin of the splitting of the PCC and GT strains.

## 3. Experimental Section

### 3.1. Strain and DNA Extraction

The GT-G strain of *Synechocystis* sp. PCC 6803 was derived from ATCC 27184. It was cultured in BG11 medium with 20 mM HEPES-NaOH (pH 7.5) at 29 °C, and illuminated with 30 μE·m^−2^·s^−1^. The cells of mid-logarithmic phase (OD_730_ = 1.0) were harvested by centrifugation at 5000× *g* for 5 min. Total DNA was extracted using the extraction kit (Dongsheng, Guangzhou, China) according to the manufacturer’s instructions.

### 3.2. Sequencing Methods and Data Analysis

The DNA was randomly fragmented by ultrasonication. Gel size selection, adaptor ligation, and amplification resulted in sequencing libraries of DNA clusters at around 300 bp. Paired-end sequencing was performed at the Illumina HiSeq 2000 flatform. The high quality sequencing data were mapped to the GT-Kasuza reference sequences using BWA software [20]. The accession numbers of reference sequences are chromosome, BA000022; pSYSM, AP004310; pSYSX, AP006585; pSYSA, AP004311; pSYSG, AP004312; pCC5.2, CP003272; pCA2.4, CP003270 and pCB2.4, CP003271. SNPs and indels were detected by VarScan software [21,22]. Large structure variation was detected by Break Dancer software [30].

### 3.3. Mutation Verification

All the putative SNPs and indels were verified through Sanger sequencing the PCR products, which covered the variation site. Annotation information was obtained from Cyanobase [31]. The reported error of database not called by software was checked by Sanger sequencing.

### 3.4. Electron Microscopy and Motility Assay

The electron microscopy and phototactic assay were performed as previously described [29]. Briefly, the cell surface structures were examined after staining with 0.8% (*w*/*v*) phosphotungstic acid (pH 7.0) under transmission electron microscope (1200EX, JEOL, Tokyo, Japan). Phototactic movement was observed on 0.8% (*w*/*v*) agar under lateral illumination. 

## 4. Conclusions

Re-sequencing of the GT-G strain of *Synechocystis* 6803 identified eight novel variants, which are likely to affect gene function. Mutations found in GT-G strain indicate that it is divergent from ATCC 27184 after the 1 bp insertion in spkA, and before the 154 bp deletion upstream and within *slr2031*. Agreement with previously reported error of database and the successful verification of variations by Sanger sequencing indicate the effectiveness and powerfulness of re-sequencing at about 200-fold coverage. Our data highlight the specific variants in the GT-G strain originated from ATCC 27184 and provide background information for future research based on GT-G strain. It also provides further evidence to identify the evolution and divergence of *Synechocystis* 6803 globally.

## Supplementary Materials

Supplementary materials can be found at http://www.mdpi.com/1422-0067/16/10/24081/s1.

## References

[1] Oliver J.W., Machado I.M., Yoneda H., Atsumi S. (2013). Cyanobacterial conversion of carbon dioxide to 2,3-butanediol. Proc. Natl. Acad. Sci. USA.

[2] Osanai T., Oikawa A., Numata K., Kuwahara A., Iijima H., Doi Y., Saito K., Hirai M.Y. (2014). Pathway-level acceleration of glycogen catabolism by a response regulator in the cyanobacterium *Synechocystis* species PCC 6803. Plant Physiol..

[3] Liu Z.X., Li H.C., Wei Y.P., Chu W.Y., Chong Y.L., Long X.H., Liu Z.P., Qin S., Shao H.B. (2015). Signal transduction pathways in *Synechocystis* sp. PCC 6803 and biotechnological implications under abiotic stress. Crit. Rev. Biotechnol..

[4] McKinlay J.B., Harwood C.S. (2010). Photobiological production of hydrogen gas as a biofuel. Curr. Opin. Biotechnol..

[5] Ikeuchi M., Tabata S. (2001). *Synechocystis* sp PCC 6803—A useful tool in the study of the genetics of cyanobacteria. Photosynth. Res..

[6] Stanier R.Y., Kunisawa R., Mandel M., Cohen-Bazire G. (1971). Purification and properties of unicellular blue-green algae (order Chroococcales). Bacteriol. Rev..

[7] Williams J.G.K. (1988). Construction of specific mutations in photosystem II photosynthetic reaction center by genetic engineering methods in *Synechocystis* 6803. Methods Enzymol..

[8] Kaneko T., Tanaka A., Sato S., Kotani H., Sazuka T., Miyajima N., Sugiura M., Tabata S. (1995). Sequence analysis of the genome of the unicellular cyanobacterium *Synechocystis* sp. strain PCC 6803. I. Sequence features in the 1 Mb region from map positions 64% to 92% of the genome. DNA Res..

[9] Kaneko T., Sato S., Kotani H., Tanaka A., Asamizu E., Nakamura Y., Miyajima N., Hirosawa M., Sugiura M., Sasamoto S. (1996). Sequence analysis of the genome of the unicellular cyanobacterium *Synechocystis* sp. strain PCC 6803. II. Sequence determination of the entire genome and assignment of potential protein-coding regions. DNA Res..

[10] Tajima N., Sato S., Maruyama F., Kaneko T., Sasaki N.V., Kurokawa K., Ohta H., Kanesaki Y., Yoshikawa H., Tabata S. (2011). Genomic structure of the cyanobacterium *Synechocystis* sp. PCC 6803 strain GT-S. DNA Res..

[11] Kanesaki Y., Shiwa Y., Tajima N., Suzuki M., Watanabe S., Sato N., Ikeuchi M., Yoshikawa H. (2012). Identification of substrain-specific mutations by massively parallel whole-genome resequencing of *Synechocystis* sp. PCC 6803. DNA Res..

[12] Trautmann D., Voss B., Wilde A., Al-Babili S., Hess W.R. (2012). Microevolution in cyanobacteria: Re-sequencing a motile substrain of *Synechocystis* sp. PCC 6803. DNA Res..

[13] Morris J.N., Crawford T.S., Jeffs A., Stockwell P.A., Eaton-Rye J.J., Summerfield T.C. (2014). Whole genome re-sequencing of two ‘wild-type’ strains of the model cyanobacterium *Synechocystis* sp. PCC 6803. N. Z. J. Bot..

[14] Tillich U.M., Wolter N., Franke P., Duehring U., Frohme M. (2014). Screening and genetic characterization of thermo-tolerant *Synechocystis* sp. PCC 6803 strains created by adaptive evolution. BMC Biotechnol..

[15] Uchiyama J., Kanesaki Y., Iwata N., Asakura R., Funamizu K., Tasaki R., Agatsuma M., Tahara H., Matsuhashi A., Yoshikawa H. (2015). Genomic analysis of parallel-evolved cyanobacterium *Synechocystis* sp. PCC 6803 under acid stress. Photosynth. Res..

[16] Zhang X., Chen G., Qin C., Wang Y., Wei D. (2012). *Slr*0643, an S2P homologue, is essential for acid acclimation in the cyanobacterium *Synechocystis* sp. PCC 6803. Microbiology.

[17] Lei H., Chen G., Wang Y., Ding Q., Wei D. (2014). *Sll*0528, a site-2-protease, is critically involved in cold, salt and hyperosmotic stress acclimation of cyanobacterium *Synechocystis* sp. PCC 6803. Int. J. Mol. Sci..

[18] Zhong L., Chen G., Ren D. (2009). Construction of *sll*0862 or *slr*0643 disrupted mutants and their phenotype analysis in *Synechocystis* sp. PCC 6803. Wei Sheng Wu Xue Bao.

[19] Chen G., Zhang X. (2010). New insights into S2P signaling cascades: Regulation, variation, and conservation. Protein Sci..

[20] Li H., Durbin R. (2009). Fast and accurate short read alignment with Burrows-Wheeler transform. Bioinformatics.

[21] Koboldt D.C., Chen K., Wylie T., Larson D.E., McLellan M.D., Mardis E.R., Weinstock G.M., Wilson R.K., Ding L. (2009). VarScan: Variant detection in massively parallel sequencing of individual and pooled samples. Bioinformatics.

[22] Koboldt D.C., Zhang Q., Larson D.E., Shen D., McLellan M.D., Lin L., Miller C.A., Mardis E.R., Ding L., Wilson R.K. (2012). VarScan 2: Somatic mutation and copy number alteration discovery in cancer by exome sequencing. Genome Res..

[23] Kamei A., Yuasa T., Orikawa K., Geng X.X., Ikeuchi M. (2001). A eukaryotic-type protein kinase, SpkA, is required for normal motility of the unicellular cyanobacterium *Synechocystis* sp strain PCC 6803. J. Bacteriol..

[24] Panichkin V.B., Arakawa-Kobayashi S., Kanaseki T., Suzuki I., Los D.A., Shestakov S.V., Murata N. (2006). Serine/threonine protein kinase SpkA in *Synechocystis* sp. strain PCC 6803 is a regulator of expression of three putative pil4 operons, formation of thick pili, and cell motility. J. Bacteriol..

[25] Mitschke J., Georg J., Scholz I., Sharma C.M., Dienst D., Bantscheff J., Voss B., Steglich C., Wilde A., Vogel J., Hess W.R. (2011). An experimentally anchored map of transcriptional start sites in the model cyanobacterium *Synechocystis* sp. PCC 6803. Proc. Natl. Acad. Sci. USA.

[26] Bhaya D., Bianco N.R., Bryant D., Grossman A. (2000). Type IV pilus biogenesis and motility in the cyanobacterium *Synechocystis* sp. PCC 6803. Mol. Microbiol..

[27] de Alda J., Houmard J. (2000). Genomic survey of cAMP and cGMP signalling components in the cyanobacterium *Synechocystis* PCC 6803. Microbiology.

[28] Liu J., Chen L., Wang J., Qiao J., Zhang W. (2012). Proteomic analysis reveals resistance mechanism against biofuel hexane in *Synechocystis* sp. PCC 6803. Biotechnol. Biofuels.

[29] Yoshihara S., Geng X., Ikeuchi M. (2002). *pilG* gene cluster and split pilL genes involved in pilus biogenesis, motility and genetic transformation in the cyanobacterium *Synechocystis* sp. PCC 6803. Plant Cell Physiol..

[30] Chen K., Wallis J.W., McLellan M.D., Larson D.E., Kalicki J.M., Pohl C.S., McGrath S.D., Wendl M.C., Zhang Q., Locke D.P. (2009). BreakDancer: An algorithm for high-resolution mapping of genomic structural variation. Nat. Methods.

[31] Cyanobase. http://genome.microbedb.jp/cyanobase/Synechocystis.

