# Supplementary material for: Identification of Specific Variations in a Non-Motile Strain of Cyanobacterium Synechocystis sp. PCC 6803 Originated from ATCC 27184 by Whole Genome Resequencing
Source: Int J Mol Sci. 2015 Oct 12;16(10):24081–93. doi: 10.3390/ijms161024081 (PMC4632739; doi:10.3390/ijms161024081)
Supplement: Supplementary file 1 [file ijms-16-24081-s001.pdf]

Supplementary Information

Table S1. Variations identified in various sequenced stains of *Synechocystis* sp. PCC 6803.

Locus	Type	GT-Kazusa Database	GT-Kazusa	GT-S	GT-I	GT-O1	GT-O2	GT-G	PCC-P	PCC-N	PCC-M	Gene ID	Annotation and Comment
Chromosome													
69849	SNP	G	G	G	G				G	A		slr1119	
125218	SNP	G	G	G	G				A	G		sll0698	hik33
125262	InDel	+	+	+	+				+	−		sll0698	hik33
126257	SNP	C	C	C	C				T	T		sll0698	hik33
144507	SNP	A									G	slr0242	bcp
386410	InDel	−	−	−	+	+	+	+	+	+	+	slr1084	
387006	SNP	C	C	C	T				C	C		slr1085	
489109	SNP	T									C	slr1609	long chain fatty acid CoA ligase
489211	SNP	G					A					slr1609	long chain fatty acid CoA ligase
509098	SNP	G					A					slr1055	chlH
731367	InDel	T	T	T	T			T	−	−	−	sll1574	T insertion cause spilt of spkA gene
781625	InDel	−	−	−	−			+	+	+	+	IGR_slr2030_slr2031	
831647	SNP	C	C	C	C	C	C	T	T	T	T	IGR ssl3441/sll1815	infA/adk
848078	SNP	G									A	slr1898	argB
842060	SNP	C	C	C	T				C	C		sll1799	rplC
886384	InDel	G				G	−					slr1204	htrA
909360	SNP	C	C	C	T				C	C		sll1968	pmgA
943495	SNP	G									A	slr1834	psaA
1070839	SNP	T									A	sll1359	
1200294	InDel	+	+	−	−	−	−	−	−	−	−	sll1780	ISY203b
1204616	SNP	G	G	G	G				A	A	A	slr1865	
1300941	InDel	+	+	+	+				−	−		slr1819	
1392586	SNP	T	T	T	C				T	T		slr1250	pstB
1423340	InDel	−	−	−	−				A	A		sll1951	insertion cause early N1438 Stop codon
1425469	InDel	A									−	sll1951	deletion cause frameshift, protein truncated
1437136	SNP	G	G	G	G				A	G		slr1992	

Table S1. *Cont.*

Locus	Type	GT-Kazusa Database	GT-Kazusa	GT-S	GT-I	GT-O1	GT-O2	GT-G	PCC-P	PCC-N	PCC-M	Gene ID	Annotation and Comment
1437389	SNP	A	A	A	A				G	G		slr1993	
1470212	SNP	G	G	G	A				G	G		sll1605	fabZ
1581467	SNP	G				A	A					sll1428	
1597057	SNP	T	T	T	T				T	G		slr1510	plsX
1763998	SNP	G	G	G	G				G	C		slr1962	
1764198	SNP	T	T	T	G				T	T		slr1962	
1765792	InDel	−						T				sll1895	
1812419	SNP	C	C	C	C				T	T	T	slr1983	
1819782	SNP	A	G	G		G	G	G				sll1876	
1819788	SNP	A	G	G		G	G	G				sll1876	
2048412	InDel	+	+	+	−	−	−	−	−	−	−	slr1635	ISY203e
2204584	InDel	G	G	−	−	−	−	−	−	−	−	slr0162	
2235441	SNP	A						G				sll1851	
2272418	SNP	C						A				slr0322	
2272927	InDel	+						−				slr0322	
2337531	SNP	G				G	A					sll0154	
2370197	SNP	A	A	A	A				A	G		slr0370	gabD
2400722	SNP	C									A	IGR sll0771/slr0774	before sll0771 (glcP)
2422495	SNP	G				G	A					sll0750	
2521013	SNP	T	T	T	T				C	C	C	slr0222	
2580625	SNP	T	T	T	T				T	A		IGR ssl0105/sll0063	
2580626	SNP	A	A	A	A				A	G		IGR ssl0105/sll0063	
2674108	SNP	C	C	C	C				T	C		slr0645	
2736514	InDel	−	−	−	−				T	T		sll0182	
2807666	SNP	C				T	T					sll0550	
2881614	InDel	−	−	−	−				−	T		slr0079	gspE
3014665	SNP	T	T	T	T	T	T	C	C	C	C	slr0302	pleD/like protein
3096187	SNP	T	C	C	T/C[a]	C	C	C	C	C	T	ssr1175	

Table S1. *Cont.*

Locus	Type	GT-Kazusa Database	GT-Kazusa	GT-S	GT-I	GT-O1	GT-O2	GT-G	PCC-P	PCC-N	PCC-M	Gene ID	Annotation and Comment
3098707	SNP	T	T	T	T	T	T	C	C	C	C	ssr1176	
3110189	SNP	G	A	A		A	A	A			A	IGR sll0665/sll0666	
3110343	SNP	G	T	T		T	T	T				sll0665	
3194022	InDel	−									A	IGR slr0533/4	
3364288	InDel	A									−	sll1496	
3371938	SNP	T									A	slr1564	
3400331	InDel	+	+	−	−	−	−	−	−	−	−	sll1474	ISY203g
3423372	SNP	C									T	slr0753	
							pSYSM						
117269	InDel	+						−			−	sll5131	ISY203j
							pSYSX						
4241	SNP	C						G				slr6004	
4253	SNP	C						T				slr6004	
4295	SNP	T						C				slr6004	
82405	SNP	A				G	G	G				ssr6089	
							pCB2.4						
1211	InDel	A						−				MYO_820	

Errors of database are in grey, + and − represent with and without insertion respectively. Blank represents it is not mentioned as variation compared with GT-Kasuza database in this strain. T/C[a]: different nucleotides are found in reference and NCBI database.
